# 1406. Hepatitis B and C Prevalence in Patients with Active and Latent Tuberculosis in an Ethnically Diverse Area of London, UK

**DOI:** 10.1093/ofid/ofab466.1598

**Published:** 2021-12-04

**Authors:** Amedine Duret, Emma Thorley, Ayolola Eni-Olotu, Oishi Sikdar, Padmasayee Papineni

**Affiliations:** 1 London North West University Healthcare NHS Trust, London, England, United Kingdom; 2 Imperial College London, London, England, United Kingdom

## Abstract

**Background:**

North West London has one of the highest tuberculosis (TB) rates in the UK, at 24.8 per 10,000. The UK prevalence of hepatitis B virus (HBV) is 0.1-0.5% and for hepatitis C virus (HCV) is 0.5-1%. Chronic infection with HBV or HCV can lead to an increased risk of adverse treatment outcomes, such as drug-induced liver injury (DILI) in patients with active or latent TB. National guidelines recommend routinely screening for HBV/HCV prior to initiating TB treatment. Our objectives were to 1) evaluate the HBV/HCV screening practice in local TB clinics, 2) establish the prevalence of HBV/HCV in patients receiving TB treatment.

**Methods:**

Retrospective analysis of laboratory and medical records of patients treated for active or latent TB identified from the London TB register and clinic records from 01/01/2018 to 31/12/2020 from London North West NHS Trust.

**Results:**

1409 patients received treatment for TB during the time period of interest; 574 (40.7%) had active disease and 835 (59.3%) had latent infection. 966/1409 patients (68.56%) were screened for HBV and HCV. 55.9% of the active TB group and 77.2% of the latent infection group were tested. 66 (6.8%) patients had isolated anti-HBc positivity, 22 (2.3%) were HBV surface antigen positive and 8 (0.8%) were HCV-antibody positive. HBV surface antigens were more prevalent in active TB patients: 9/321 (2.80%) with active TB versus 13/645 (2.02%) with latent TB. 36/321 (11.21%) active TB patients had HBV core antibodies compared to 30/645 (4.65%) latent TB patients (p < 0.001). Three patients started antiviral treatment following their viral hepatitis diagnosis (one with HBV, two with HCV).

**Conclusion:**

The prevalence of chronic HBV in the study population was higher than the estimated UK prevalence. Fifteen diagnoses of hepatitis were new, allowing specialist referral for monitoring of fibrosis and development of hepatocellular carcinoma. Three patients required hepatitis treatment. 6.8% of patients were positive for anti-HBc and therefore identified as being at future risk of HBV reactivation if requiring immunosuppressive therapies.TB disproportionately affects marginalised communities; screening for viral hepatitis in TB clinic represents an opportunity to target these hard-to-reach groups to maximise the impact of public health interventions.

**Disclosures:**

**All Authors**: No reported disclosures

